# Efficacy and Mechanisms of Action of Essential Oils’ Vapours against Blue Mould on Apples Caused by *Penicillium expansum*

**DOI:** 10.3390/ijms24032900

**Published:** 2023-02-02

**Authors:** Fabio Buonsenso, Giada Schiavon, Davide Spadaro

**Affiliations:** 1 Department of Agricultural, Forestry and Food Sciences (DISAFA), University of Turin, Largo Paolo Braccini 2, 10095 Grugliasco, Italy; 2 Centre of Competence for the Innovation in the Agro-Environmental Sector—AGROINNOVA, University of Turin, Largo Paolo Braccini 2, 10095 Grugliasco, Italy

**Keywords:** biofumigation, natural antifungal compounds, antimicrobial, antioxidant, postharvest disease, sustainable agriculture, *Malus x domestica*, quality factors, gas chromatography analysis, SPME-GC-MS

## Abstract

Biofumigation with slow-release diffusers of essential oils (EOs) of basil, oregano, savoury, thyme, lemon, and fennel was assessed for the control of blue mould of apples, caused by *Penicillium expansum*. In vitro, the ability of the six EOs to inhibit the mycelial growth was evaluated at concentrations of 1.0, 0.5, and 0.1%. EOs of thyme, savoury, and oregano, at all three concentrations, and basil, at 1.0 and 0.5%, were effective in inhibiting the mycelial growth of *P. expansum*. In vivo, disease incidence and severity were evaluated on ‘Opal’ apples artificially inoculated with the pathogen and treated at concentrations of 1.0% and 0.5% of EOs. The highest efficacy in reducing blue mould was observed with EOs of lemon and oregano at 1.0% after 60 days of storage at 1 ± 1 °C (incidence of rot, 3 and 1%, respectively) and after a further 14 days of shelf-life at 15 ± 1 °C (15 and 17%). Firmness, titratable acidity, and total soluble solids were evaluated at harvest, after cold storage, and after shelf-life. Throughout the storage period, no evident phytotoxic effects were observed. The EOs used were characterised through GC-MS to analyse their compositions. Moreover, the volatile organic compounds (VOCs) present in the cabinets were characterised during storage using the SPME-GC-MS technique. The antifungal effects of EOs were confirmed both in vitro and in vivo and the possible mechanisms of action were hypothesised. High concentrations of antimicrobial and antioxidant compounds in the EOs explain the efficacy of biofumigation in postharvest disease control. These findings provide new insights for the development of sustainable strategies for the management of postharvest diseases and the reduction of fruit losses during storage.

## 1. Introduction

Apples (*Malus x domestica* Borkhausen) are one of the most important fruit products in the European Union trade, where apples represent 29% of the fruit production [1]. Apples are also among the five most traded fruits in Europe, with a commercial value of 2.27 billion euros in 2020. Over 600 million euros of apples were imported from non-European countries, mainly counter-seasonal from the southern hemisphere [2,3].

Postharvest disease of apples, such as blue mould (caused by *Penicillium expansum*), grey mould (*Botrytis cinerea*), bitter rot (*Colletotrichum* spp.), bull’s eye rot (*Neofabraea* spp.), apple scab (*Venturia inaequalis*), or dry lenticel rot (*Ramularia mali*), represent a significant obstacle to security and safe access to these foods, with resulting economic damages and risks to human health [4,5,6,7,8].

*Penicillium expansum* Link is one of the most relevant postharvest pathogens of apples [9]. The pathogen causes blue mould, which starts with watery and soft lesions, light brown in colour, and blue-green conidia [10,11]. Blue mould negatively affects fruit quality and significantly contributes to food losses. Its danger to human health is due to the biosynthesis of important secondary metabolites, such as chaetoglobosins, citrinin, communesins, expansolides A and B, andrastin, ochratoxin A, patulin, penitrem A, roquefortine C, and rubratoxin B [12,13,14,15]. In particular, the mycotoxins patulin and citrinin are responsible for genotoxic, immunosuppressive, neurological, gastrointestinal toxic effects (patulin), and nephrotoxic effects (citrinin) [16,17,18].

*P. expansum* grows very well in a temperature range between −2 and 35 °C. Therefore, storing apples in cold rooms is not sufficient, or is ineffective, to prevent and control blue mould development [19,20,21]. Over the years, numerous strategies have been developed to control the postharvest pathogen. Among these, the use of fungicides must certainly be mentioned, but the number of fungicides available for postharvest treatment is limited, and several strains of *Penicillium* spp. resistant to fungicides have been reported [22,23,24,25]. For these reasons, along with environmental and health considerations, new innovative and sustainable approaches and tools are needed to control blue mould during storage and consequently preserve the quality of the fruit and reduce postharvest losses. Antifungal techniques, such as ozone treatment [10], use of biocontrol agents (BCA) [26,27,28], and the use of natural products with intrinsic antifungal activities [29,30,31,32], represent valid alternatives to synthetic fungicides.

Previous works showed that biofumigation with essential oils (EOs) could be useful to inhibit the fungal infection and to reduce disease development during postharvest storage [33,34,35]. EOs represent a powerful tool to reduce the environmental footprint of fruit storage [36,37]. The antifungal activity of EOs strictly depends on their chemical composition [38,39]. In fact, molecules with a phenolic structure, for example thymol and carvacrol, or with aldehydes, such as p-anisaldehyde and ketones, significantly inhibit the pathogen growth. The above-mentioned molecules have proven fungicidal activity, and EOs rich in these components showed the highest inhibitory activity against many postharvest pathogens [40,41]. The EOs of thyme (*Thymus vulgaris*) and savoury (*Satureja montana*), mainly composed of thymol and carvacrol, are highly effective in the control of fungal pathogens [33]. In general, the effectiveness of EOs is studied through direct contact with fruit, achieved by spraying EO emulsions on the fruit or by dipping the fruit in the EO emulsion [42]. However, these application methods can show undesirable effects, such as phytotoxicity. In fact, treatments with EOs at 10% (*v*/*v*) were highly phytotoxic. The lesions developed were proportional to the efficacy shown by the treatments with the essential oils’ concentration [37]. Additionally, the use of EOs can lead to alterations in the organoleptic characteristics of the fruit, such as changes in taste or flavour after cold storage [35,43]. In the latter case, exposure for at least 12 h after storage could greatly reduce unpleasant flavours. Therefore, the development of different methodologies for applying EOs on apples, such as biofumigation, is necessary. 

Biofumigation with EOs of thyme and savoury, characterised by a strong antifungal activity, led to effective control of various postharvest pathogens, such as *Aspergillus* spp., *Penicillium* spp., and *Trichoderma* spp. [44,45,46]. This application method avoids direct contact with the fruit skin, and consequently, alteration of the sensory profile and phytotoxicity are limited. In addition, EO vapours show positive effects in maintaining nutritional values, avoiding the oxidation of ascorbic acid and carotenoids, as demonstrated on peaches and nectarines [34].

The aim of this work was to evaluate the efficacy of six different EOs applied by biofumigation in controlling blue mould caused by *P. expansum* on ‘Opal’ apples. The EOs from basil (*Ocimum basilicum*), savoury (*Satureja montana*), oregano (*Origanum vulgare*), thyme (*Thymus vulgaris*), fennel (*Foeniculum vulgare dulce*), and lemon (*Citrus limon*) were tested at different concentrations. A further aim was to measure the occurrence of the major components of EOs through sampling of volatile components during two months of storage in cabinets, under controlled conditions, using solid-phase micro-extraction and a gas chromatography system coupled with mass spectrometry (SPME-GC-MS).

## 2. Results and Discussion

### 2.1. In Vitro Efficacy Tests against P. expansum

In order to clarify the composition, the six essential oils were analysed through the gas chromatography technique coupled with mass spectrometry (GC-MS). In the Supplementary Materials (Table S1), the composition of EOs and the retention times (expressed in minutes) of the individual molecules are reported. The major component in the EOs of oregano and savoury is carvacrol (68.0% and 41.5%, respectively). In the thyme EO, the major component is thymol (43.3%). In basil, there is a high percentage of linalool (58.3%), while for lemon and fennel the major components are limonene (66.9%) and trans-anethole (50.5%), respectively.

**Figure 1:**
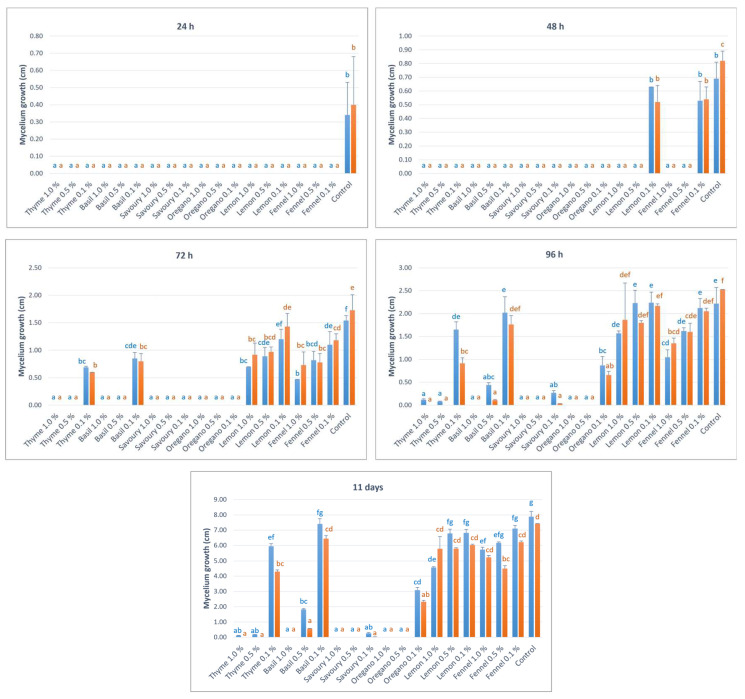
Effect of volatile organic compounds released by EOs on the growth of the mycelium (diameter, cm) of *P. expansum* (two strains, PEN2, blue bars, and PEN3, orange bars) through the sandwich plates method. Essential oils were applied at different concentrations (0.1%, 0.5%, and 1.0%). The plates were cultured at 25 °C and the measurements were carried out after 24 h, 48 h, 72 h, 96 h, and 11 days. Values of the same strain and time point, followed by the same letter, are not statistically different according to the Tukey’s test (*p* < 0.05).

The antifungal activity of EOs of basil, oregano, savoury, thyme, fennel, and lemon at concentrations of 1.0%, 0.5%, and 0.1% was evaluated in vitro, by measuring the inhibition of the mycelial growth of two strains of *P. expansum* (PEN2 and PEN3). As can be seen in Figure 1, although after 24 h all essential oils at all three concentrations evaluated were statistically effective in inhibiting the mycelial growth of *P. expansum* compared to the control, a situation that extends to both strains evaluated, the same cannot be said if the statistical data were observed after 96 h. In fact, the EOs of lemon, at all three concentrations tested, as well as of fennel, at 0.5 and 0.1%, and of basil at 0.1%, were not statistically different from the control. 

Among the different EOs used against blue mould, thyme, basil, savoury, and oregano had antimicrobial activity, inhibiting from ~71% to ~100% of the growth of the fungus in vitro at concentrations of 1.0% and 0.5% after 11 days (Figure 1). Only savoury EO showed significant efficacy even at a concentration of 0.1%, with an inhibition of mycelium growth greater than 96%.

### 2.2. Efficacy of EOs against Blue Mould on Apples and Effect on Fruit Quality

**Figure 2:**
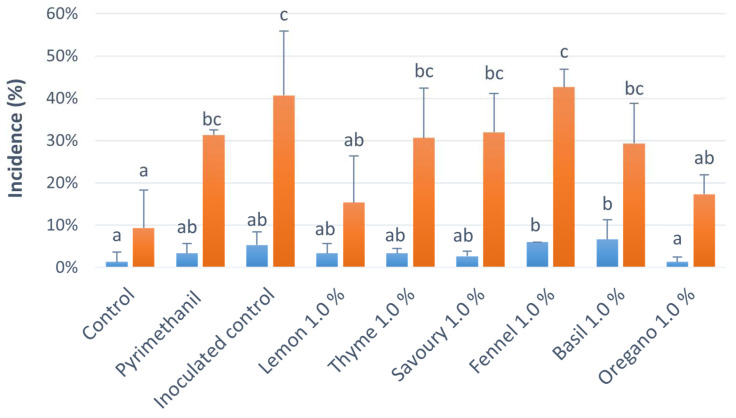
Incidence of rot on ‘Opal’ apples treated with EOs’ biofumigation after 60 days of storage at 1 ± 1 °C (blue bars) and 95% relative humidity, and after 14 days of shelf-life at 15 ± 1 °C (orange bars). Values of the same time point, followed by the same letter, are not statistically different according to the Tukey’s test (*p* < 0.05).

A trial was performed to evaluate the effect of the application of the EOs at 1.0% through biofumigation on blue mould incidence on ‘Opal’ apples. After 30 days of storage, the fruit showed no rot. The graph in Figure 2 (and Table S10, Supplementary Materials) shows the disease incidence after 60 days of storage and after 14 days of shelf-life. After 60 days of storage, all the EOs significantly reduced blue mould on apple, but the oregano EO at 1.0% was the most effective treatment. After 14 days of shelf-life, the EOs of lemon 1.0% and oregano 1.0% were the most effective in the control of blue mould and the only ones with a significant reduction compared to the inoculated control.

The growth of *P. expansum* was significantly inhibited in vitro by the EOs of savoury at 1.0%, 0.5%, and 0.1%, and of thyme, basil, and oregano at 1.0% and 0.5% at 11 days of incubation, but the development of blue mould on apples after shelf-life was significantly reduced only by lemon and oregano EOs. The different antifungal effects of EOs in MEA and apples could be attributed to complex components and structures of the fruit host or to the interactions between *P. expansum*, the fruit microbiome, and apples [35,47], but it could also depend on the concentration of volatiles, the temperature, and the application method [48].

The quality analysis provided the ripening profile of the fruit during storage. Firmness, total soluble solids, and titratable acidity were analysed immediately after harvest, after 60 days of storage, and finally, after 14 days of shelf-life (Table 1). These data show a normal fruit ripening profile over time, with a decrease in firmness and titratable acidity and an increase in total soluble solids. 

Fruits treated with the essential oil of oregano 1.0% after 60 days of storage were significantly firmer than the inoculated control. The firmness reduction is due to the release and activity of pectinolytic enzymes. Some components of the EOs are able to decrease lipid enzymatic oxidation (LOX) by scavenging some oxidative radical species and could inhibit the enzymes that degrade the pectin, delaying the maturation, decreasing the browning and deterioration of fruits, and maintaining the firmness of the product for a longer time [49,50].

The increase of total soluble solids (TSS) during maturation is due to the starch degradation caused by hydrolases, resulting in the accumulation of sugars [51]. Fruit treated with savoury, lemon, thyme, and fennel had a statistically lower level of TSS than the inoculated control. During shelf-life, lemon and savoury maintained the lowest TSS level compared to the control, followed by the thyme essential oil treatment. The inoculation with *P. expansum* could have favoured an increase of TSS in the inoculated control compared to the healthy control, due to the rapid conversion of primary metabolites into energy required to synthesise antioxidant defence compounds against the pathogen infection [52]. Antioxidant molecules are naturally components of EOs, in particular in savoury and thyme (as show in Table S1). Treatments with these two EOs showed the lowest TSS levels favoured by the high antioxidant properties of the main components of these EOs.

A decrease in acidity is related to the sugar accumulation derived from starch degradation during fruit ripening. Organic acids reduce as they are used as substrates for the respiration process [53]. Titratable acidity, after shelf-life, showed significantly higher levels compared to the inoculated control, in most fruit treated with EOs such as lemon, thyme, basil, and oregano.

**Figure 3:**
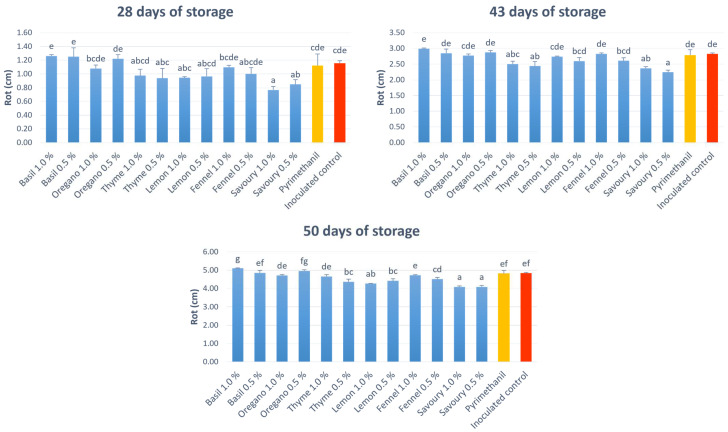
Severity of rot caused by *P. expansum* on ‘Opal’ apples treated through the biofumigation of EOs (blue bars) at 28 and 43 days, at a temperature of 1 ± 1 °C, and after 50 days, i.e., after 7 days of shelf-life at 15 ± 1 °C. Yellow bars represents apples treated with pyrimethanil, red bars represent inoculated control. Values of the same time point, followed by the same letter, are not statistically different according to the Tukey’s test (*p* < 0.05).

In a second experiment (Figure 3), disease severity data were obtained from apples artificially inoculated with *P. expansum*, biofumigated with EOs at 0.5% and 1.0%, and stored for 50 days at 1 ± 1 °C. At 28 days of storage, the only fruit with a significantly lower rot diameter compared to the inoculated control were apples treated with savoury EOs at 0.5% or 1.0%. After 43 days of storage, besides fruit treated with the savoury EO, fruit treated with the thyme EO at 0.5% and 1.0% also showed a lower disease severity compared to the inoculated control. Finally, after 50 days of storage, fruit treated with the lemon EO at 0.5% and 1.0% also had a lower rot diameter compared to the control. At the three time points, the lowest disease severity was obtained with fruit treated with the savoury EO at both concentrations. 

### 2.3. Characterisation of Volatile Compounds of EOs in the Cabinets during Storage

Together with the evaluation of blue mould incidence and severity, the volatile compounds of the EOs applied at 1.0% present in the cabinet atmosphere were characterised, through the SPME-GC-MS technique. Sampling was carried out at 1, 10, 28, and 43 days of storage and after 7 days of shelf-life (Supplementary Materials, Tables S2–S9). In addition, the atmosphere of the cabinets containing the healthy control and inoculated control fruit was sampled (Tables S2 and S3, respectively) to identify the volatile molecules released by apples, with or without *P. expansum* inoculation. Ester molecules with different numbers of carbon atoms (hexyl acetate, hexyl butyrate, hexyl 2-methylbutanoate, hexyl hexanoate, 2,2,4-trimethyl-3-(carboxyisopropyl)pentanoic acid isobutyl ester, 2,6,10,14-tetramethyl pentadecane, and α-farnesene) are naturally released during the apple ripening process. 

Data obtained from the storage cabinets (Tables S4–S9), where the six EOs were released by slow-release diffusers, showed how the molecules α-thujene, α-pinene, and camphene, and in some cases also sabinene and β-pinene, were not yet detectable after 24 h of storage. p-Cymene, present in the composition of all the essential oils analysed, was detected after 24 h at concentrations from 1.02 to 2.44 ppm for five EOs, but at 18.86 ppm for the savoury EO. Concerning the savoury EO, p-cymene amounted to values of 24.36, 25.88, 19.91, and 15.82 ppm, respectively, after 10, 28, 43, and 50 days. For the EOs of basil, oregano, thyme, and fennel, p-cymene never exceeded 7 ppm, while for limonene the maximum concentration observed at 28 days was 11.83 ppm. Linalool is also one of the components of all six essential oils analysed and it is the major component in basil EO (58.28%). By treating with basil EO, linalool was present at 42.97 ppm after 24 h, and it later decreased to 21.45 and 9.71 ppm, respectively, after 10 and 28 days. At the last two time points, its concentration remained stable: 10.95 ppm and 11.30 ppm, after 43 and 50 days. Present only in basil (1.16%) and fennel (50.45%), trans-anethole always had concentrations lower than 1 ppm in fruit treated with the basil EO, whereas its concentration ranged from 9.07 ppm at 24 h to 23.41 ppm at 43 days for fruit treated with the fennel EO. 

Both thymol and carvacrol are present in the EOs of oregano, savoury, and thyme. Thymol is the main component of thyme essential oil (43.26%), and a decrease in its concentration from 19.70 ppm at 1 day to 7.61 ppm at 43 days was observed in the cabinet atmosphere. Its concentration increased at 50 days (17.78 ppm). In the case of oregano (1.98%) and savoury (4.12%), thymol never exceeded 2.82 ppm. In oregano and savoury EOs, carvacrol represents the majority component (68% in oregano and 41.45% in savoury). In the cabinet atmosphere, a decrease from 81.41 ppm at 1 day to 28.20 ppm at 43 days was observed for oregano, whereas for savoury it ranged from 19.67 to 41.55 ppm at the first time points. For both oregano and savoury EOs, there was a significant increase of carvacrol at 50 days: 64.87 and 53.37 ppm, respectively. In the thyme EO (4.43% in composition), carvacrol never exceeded 5.93 ppm in the cabinets.

### 2.4. Evaluation of the Possible Mechanisms of Action

By considering the abundance of volatile molecules in the cabinet atmosphere, it is possible to evaluate the effect of the EOs’ components on in vitro of inhibition of the mycelial growth of *P. expansum* and on the reduction of blue mould incidence and severity on apple. The antifungal (fungistatic) and antioxidant activities of EOs may be due to the synergistic action of two or more compounds, rather than to a single characteristic molecule. The activity of EOs is expressed both through the direct inhibition of *P. expansum* mycelial growth [54,55] and through an antioxidant activity [56,57,58,59]. In the antifungal activity, the mechanism of action involves the interaction of hydrophobic compounds with the lipids of the fungal cell membrane. In addition, the involvement of ergosterol synthesis in the plasma membrane of fungi has been hypothesised. The resulting loss of membrane integrity induces changes in the electron transport chain, nutrient absorption, and in protein and nucleic acid synthesis, and it could inhibit essential enzymes for energy metabolism and coagulate the cellular content, finally causing cell death [60,61].

From EOs’ compositions, it is possible to roughly predict the antioxidant potential, important to reduce lipid oxidation and increase the fruit shelf-life [56]. An antioxidant behaviour could be predicted for EOs rich in phenolics (characterised by high reactivity with peroxyl radicals, which are eliminated by formal transfer of hydrogen atoms [57]) and poor in unsaturated terpenes. Large amounts of molecules with phenolic groups combined with the presence of components similar to cyclohexadiene, for example α-phellandrene, are likely to exhibit an even higher antioxidant activity. In contrast, the absence of these two classes of compounds is presumed to result in minimal antioxidant activity [56]. 

**Figure 4:**
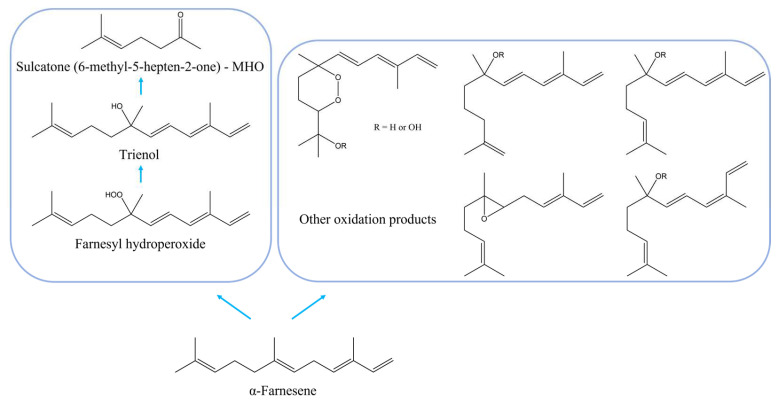
Chemical structures of α-farnesene and oxidation products.

α-Farnesene is a hydrocarbon sesquiterpene, chemically unstable and subject to easy oxidation, both in vivo and in vitro, which leads to the formation of the products illustrated in Figure 4. As shown in Table S1, α-farnesene is a component of the basil (2.15%) and lemon (0.37%) EOs. Treatment with all the tested EOs leads to different concentrations of α-farnesene during storage in the cabinet atmosphere at 1 ± 1 °C and throughout the shelf-life (Tables S2–S9) with respect to healthy and inoculated controls. The oxidation of α-farnesene is responsible for apple superficial scald [62,63,64], which represents the main postharvest physiological disorder of apples and is associated with tissue breakdown below the fruit skin [65].

Oxidation products, accumulated in apple epicuticular wax and peel tissue during cold storage, have been identified as conjugated trienols [64,65,66] and represent the real cause of the superficial scald. It has also been shown that α-farnesene is involved in the pathogenesis induced by *P. expansum* on apples [67]: an increased release of α-farnesene in fruit has been observed after fungal infection [68]. Therefore, the reduction in the release of α-farnesene [61] and the inhibition/slowdown of oxidation processes are associated with a decrease in the onset of the infection induced by *P. expansum*, which is no longer advantaged by the damage caused by the oxidation products of α-farnesene.

For this reason, the use of essential oils rich in antioxidant compounds is an excellent strategy to reduce both the fungal activity and the adverse effects due to the oxidation products of α-farnesene.

**Figure 5:**
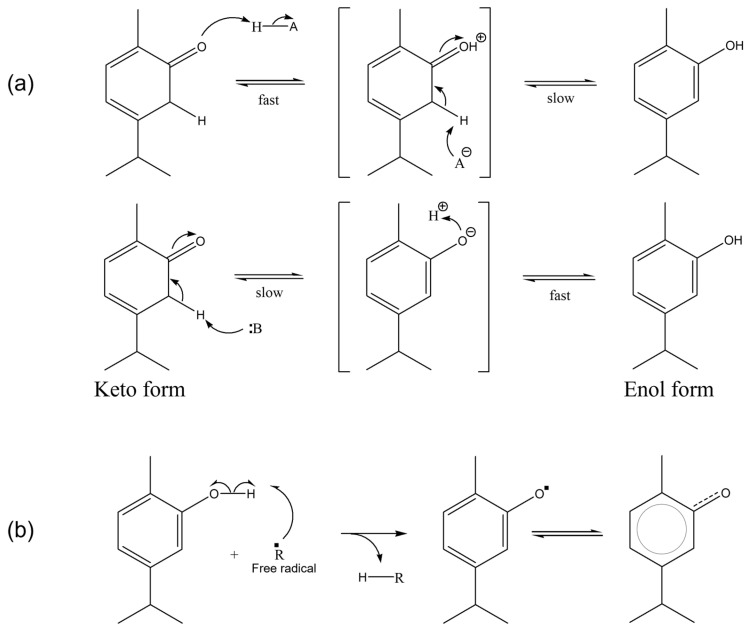
(**a**) Tautomerisation process of carvacrol, acid (top) and basic (bottom) catalysed. (**b**) Homolytic scission of the hydroxyl group of carvacrol and resonance structure.

Experimentally, thymol and carvacrol, monoterpenoid phenol compounds, showed the best radical scavenging activity [58]. Between the two molecules, however, carvacrol has a greater activity due to the presence of the hydroxyl group in the ortho position in relation to the methyl group, ensuring better delocalisation of the electron by resonance, in contrast to thymol which instead has the -OH group in the meta position [58,59,69] (Figure 5).

**Figure 6:**
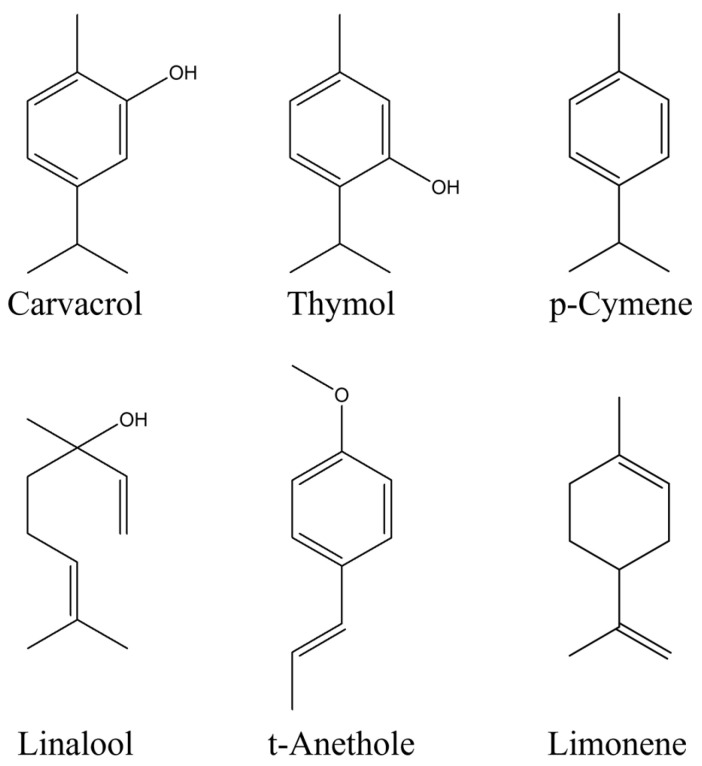
Chemical structures of carvacrol, thymol, p-cymene, linalool, t-anethole, and limonene.

Additionally, trans-anethole (Figure 6), a derivative of phenylpropene, shows marked antioxidant activity, albeit lower than carvacrol and thymol, also presenting, due to its chemical structure, the ability to add a hydroperoxyl radical.

Finally, limonene, linalool, and p-cymene (Figure 6), three monoterpenic compounds, show a low antioxidant activity. These indications are consistent with the results obtained in this work. Indeed, the use, via biofumigation, of the essential oil of savoury, composed by both carvacrol (main component) and thymol, provided the highest reduction of blue mould incidence and severity (Figure 3). Conversely, the use of basil, which has linalool as a constituent but not the phenolic derivatives carvacrol and thymol, offered the lowest reduction of disease incidence and severity (Figure 2 and Figure 3).

## 3. Materials and Methods

### 3.1. Essential Oils and Chromatographic Analysis

The EOs of basil (*Ocimum basilicum*), oregano (*Origanum vulgare*), savoury (*Satureja montana*), thyme (*Thymus vulgaris*), lemon (*Citrus limon*), and fennel (*Foeniculum vulgare dulce*) used in the experiments were purchased from Flora s.r.l. (Lorenzana (PI), Italy; Certifications quality assurance: UNI EN ISO 9001 and 14001). The compositional analysis was performed using a gas chromatograph, Shimadzu GC-2010 Plus (Shimadzu, Kyoto, Japan), equipped with a mass spectrometer, GCMS-QP 2010 Ultra (Shimadzu), and a split–splitless injector. The gas chromatograph was fitted with a Zebron ZB-5MSi (Phenomenex, Torrance, CA, USA) fused silica capillary column (30 m × 0.25 mm L × ID) with a 0.25 µm film thickness. Helium carrier gas using a linear velocity of 36.7 cm/s with a constant flow rate of 1.0 mL/min was used. The pressure was 61.3 kPa and total flow was 84 mL/min for split mode (split ratio: 80.0) and 4 mL/min for splitless mode. Ion electron impact spectra at 70 eV were recorded in scan mode (35–700 *m*/*z*). For all EOs, the oven program started with an initial temperature of 70 °C for 3 min, heating at 2 °C/min to 150 °C, 150 °C for 10 min, heating at 2 °C/min to 220 °C, 220 °C for 5 min, heating at 20 °C/min to 280 °C, and, finally, held for 2 min. The injection temperature was set at 250 °C and the ion source and the interface were both set at 280 °C. Pure EOs were diluted at 1.0% in n-hexane HPLC grade (VWR, Radnor, PA, USA) for direct injection using split mode (split ratio: 80.0). Cabinet sampling was performed using SPME fibre assembly polydimethylsiloxane (PDMS) df 30 μm, fused silica needle size 24 Ga, for use with a manual holder, 3pk (Supelco Analytical, Bellefonte, PA, USA), for 10 min in quintuplicate (at 1, 10, 28, and 43 days of storage at 1 ± 1 °C, and after 7 days of shelf-life at 15 ± 1 °C), whereby the injector was in splitless mode. Sampling was performed for treatments with the six essential oils, each one at a 1.0% concentration. After extraction, SPME was introduced into the heated injector port of the chromatograph for desorption at 250 °C for 30 s. The relative composition (percentage) of volatile compounds was calculated by comparing the peak area to the area of the total chromatogram (from 5 to 50 min). Absolute quantification was calculated for carvacrol (R^2^ = 0.9972; LOD: 4.15 ppm, LOQ: 12.57 ppm), thymol (R^2^ = 0.9999; LOD: 0.93 ppm, LOQ: 2.82 ppm), linalool (R^2^ = 0.9955; LOD: 1.00 ppm, LOQ: 3.03 ppm), anethole (R^2^ = 0.9996; LOD: 3.11 ppm, LOQ: 9.41 ppm), limonene (R^2^ = 0.9967; LOD: 4.01 ppm, LOQ: 12.16 ppm), and p-cymene (R^2^ = 0.9955; LOD: 4.85 ppm, LOQ: 14.71 ppm) using a standard calibration curve between 1 and 75 ppm (mg/L). Relative quantification was determined for the other compounds by using the standard calibration curve of the above-mentioned molecules.

### 3.2. Antifungal Tests In Vitro

The antifungal activity of EOs of basil, savoury, thyme, oregano, lemon, and fennel at concentrations of 1.0%, 0.5%, and 0.1% was evaluated in vitro, using the sandwich plates method, to evaluate the mycelial growth inhibition of two strains (PEN2 and PEN3) of *P. expansum*, taken from the collection of the University of Turin and isolated from apples. For the preparation of the EOs, Petri dishes with a diameter of 90 mm (VWR, Milan, Italy) were used, where Potato Dextrose Agar (PDA, Merck, Darmstadt, Germany) medium was poured, after autoclaving, with the selected EO. The EO was added at 1.0%, 0.5%, or 0.1% (*v*/*v*) to sterile deionised water (98.0, 98.5, or 98.9% *v*/*v*) and Tween 20 (1.0% *v*/*v*). For the pathogen growth, Malt Extract Agar (MEA, Merck) plates were inoculated with mycelium plugs (0.4 cm) taken from cultures grown on MEA for 21 days. Plates with the EOs were placed on top of plates with the mycelium plug to build a sandwich and then closed with parafilm. The plates were incubated at 25 ± 1 °C and the mycelium diameter of the pathogen was measured after 24 h, 48 h, 72 h, 96 h, and 11 days of incubation. Control plates were set up in the same manner as described above but using a plate containing only PDA. The test was performed twice, each time with 5 biological replicates.

### 3.3. Efficacy against Blue Mould on Apples

To evaluate the effect of the EOs on the disease incidence, the trial was set up using the six EOs at 1.0%. ‘Opal’ apples, purchased from Ortofruit Italia (Saluzzo, Italy), were selected based on absence of injuries and size homogeneity. Fruits were artificially inoculated with two strains of *P. expansum* (PEN2 and PEN3), taken from the collection of the University of Turin and isolated from ‘Opal’ apples. Three control treatments were prepared: the chemical control inoculated with *P. expansum* and treated with the fungicide pyrimethanil (Scala, a.i.: 36.8%, BASF, Ludwigshafen, Germany) at a 0.067% concentration, the inoculated control with *P. expansum*, and a healthy control, not inoculated. ‘Opal’ apples were inoculated with a conidial suspension of *P. expansum*. For the preparation of the conidial suspension, two *P. expansum* strains were cultured on PDA added with 0.025 g of streptomycin (AppliChem, Darmstadt, Germany) for 5 days. The plates contained 39 g of PDA (Merck) and 4 g of agar (Merck) per litre of deionised water (dH_2_O). Conidia were collected with a Drigalsky spatula, and a 1.0% solution of the Tween-20 surfactant and conidial suspension was brought to the concentration of 1 × 10^5^ conidia/mL in a Burker chamber. Apples were inoculated by immersion in the conidial suspension for 1 min, then the fruits were air-dried for 3 h before being placed in the cabinets. The treatment with the EOs was carried out by biofumigation, placing six slow-release diffusers inside each cabinet. Each diffuser was a Petri dish of 90 mm-diameter containing 1 mL of EO and 1 mL of Tween-20 incorporated into 98 mL of Water Agar (WA), after autoclaving. The trial was carried out in storage cabinets kept at 1 ± 1 °C and 95% relative humidity for 60 days, followed by a 14-day shelf-life period at 15 ± 1 °C. For the trial, 1350 fruits were used: 3 boxes of 50 fruit were used per treatment. The cabinet atmosphere was sampled using the SPME-GC-MS technique throughout the experiment.

Disease severity data were obtained from apples artificially inoculated with the strains PEN2 and PEN3 of *P. expansum*. A wound of about 2 mm was made on apples where a conidial suspension of *P. expansum* was inoculated. Fruits were placed in cabinets with biofumigation plates containing the EOs at 0.5% and 1.0%. The rot diameter was measured after 28 and 43 days of storage at 1 ± 1 °C and after a further 7 days of shelf-life at 15 ± 1 °C. The diameter of the lesion carried out for the inoculation equal to 0.2 cm was subtracted from the value obtained. For the trial, 630 fruit were used: each treatment included 3 boxes of 15 apples.

### 3.4. Effect on Fruit Quality

Fruit quality analysis were performed by measuring firmness, total soluble solids (TSS), and titratable acidity on 3 replicates of 4 fruit per time point and treatment. Fruit quality parameters were measured at harvest, at the end of storage, and at the end of the shelf-life.

Firmness was determined using the Fruit Texture Analyzer (FTA, Turoni, Italy) with an 11 mm tip on two opposite points of the fruit surface. The firmness data (N/cm^2^) were measured on two points of the equatorial region of the apples.

To determine the TSS, a NR151 refractometer (Rose Scientific Ltd., Edmonton, AB, Canada) was used. The values obtained (degrees Brix) from the measurement are expressed as a percentage of total soluble solids content. The juice was extracted from four fruits per replicate, in three replicates.

The titratable acidity was determined using 6 g of juice obtained by cutting the fruit at room temperature in an extractor. For each sample, 6 g of juice was weighed and added to 50 mL of water. Each sample was titrated with sodium hydroxide (NaOH 0.1 N) up to a final pH value of 8.2 using the Five Easy Plus pH meter FP20-Std-Kit (Mettler Toledo, Milan, Italy). The titratable acidity was calculated using the following equation:

(V_NaOH_ × 0.0067 × 100)/6,

where 0.0067 indicates the acidity factor of the malic acid and the value 6 represents, instead, the grams of juice analysed.

### 3.5. Statistical Analysis

Statistical analysis was performed by one-way analysis of variance (ANOVA). Statistical significance was judged at the level of *p*-value < 0.05. When the analysis of variance was statistically significant, Tukey’s test was used to separate the means. Data were analysed using IBM SPSS Statistics, Version: 28.0.1.0 (142) (IBM Corp., Armonk, NY, USA).

## 4. Conclusions

Savoury, oregano, thyme, and basil essential oils’ vapours have shown a high antifungal activity in vitro against *P. expansum,* even at low concentrations. In experiments on apples inoculated with *P. expansum*, the essential oil of savoury by biofumigation proved to be the most effective in reducing the severity of rot induced by the fungus. Instead, basil essential oil provided the lowest results, despite the inhibition ability shown with in vitro experiments. These findings may suggest that the antioxidant action of the components of essential oils plays a central role in determining their effectiveness. Furthermore, the essential oil vapours did not affect the fruit quality, including firmness, acidity, and total soluble solids. Finally, it is possible to hypothesise that the antifungal activity of essential oils was not linked to the activity of a single molecule but to the synergistic activity of two or more compounds. Different molecules may act through both direct inhibition of the fungal growth and indirect antioxidant action. In the context of the reduction of pesticides’ use, essential oils may represent a valid alternative to control postharvest diseases of fruit and vegetables.

## Figures and Tables

**Table 1:** Firmness, total soluble solids (TSS), and titratable acidity of ‘Opal’ apples treated by biofumigation with EOs. Apples were stored at 1 ± 1 °C and 95% relative humidity for 60 days and subsequently at 15 ± 1 °C for 14 days. Each value is the average of n = 3 replicates of 4 fruits. Data of the same survey, with the same letter, are not significantly different according to the Tukey’s test (*p* < 0.05).

Time Point (Temperature)	Treatment (Concentration)	Firmness (N/cm^2^) ± SD *	Total Soluble Sugar (%) ± SD *	Titratable Acidity (%) ± SD *
At harvest		91.86 ± 14.18	12.30 ± 0.10	0.50 ± 0.13
60 days (1 ± 1 °C)	Control	76.76 ± 8.97 ^ab^	14.27 ± 1.60 ^ab^	0.48 ± 0.01 ^a^
	Pyrimethanil	73.21 ± 10.49 ^ab^	14.07 ± 0.12 ^ab^	0.38 ± 0.02 ^a^
	Inoculated control	74.99 ± 15.63 ^ab^	15.50 ± 0.17 ^b^	0.36 ± 0.01 ^a^
	Lemon (1.0%)	83.08 ± 19.04 ^bc^	13.57 ± 0.23 ^a^	0.36 ± 0.11 ^a^
	Thyme (1.0%)	76.46 ± 10.28 ^ab^	13.60 ± 0.87 ^a^	0.35 ± 0.01 ^a^
	Savoury (1.0%)	75.47 ± 11.14 ^ab^	12.60 ± 1.21 ^a^	0.39 ± 0.01 ^a^
	Fennel (1.0%)	76.09 ± 15.21 ^ab^	12.70 ± 0.17 ^a^	0.39 ± 0.01 ^a^
	Basil (1.0%)	71.83 ±12.35 ^a^	15.43 ± 1.27 ^b^	0.41 ± 0.01 ^a^
	Oregano (1.0%)	86.73 ± 19.81 ^c^	13.97 ± 1.27 ^ab^	0.31 ± 0.02 ^a^
14 days of shelf-life (15 ± 1 °C)	Control	66.79 ± 14.55 ^a^	14.33 ± 1.33 ^bc^	0.28 ± 0.01 ^bc^
	Pyrimethanil	67.50 ± 11.35 ^a^	14.97 ± 0.23 ^c^	0.25 ± 0.00 ^a^
	Inoculated control	65.85 ± 12.10 ^a^	15.30 ± 0.35 ^c^	0.25 ± 0.00 ^a^
	Lemon (1.0%)	71.60 ± 15.14 ^a^	13.60 ± 0.69 ^ab^	0.32 ± 0.02 ^d^
	Thyme (1.0%)	66.55 ± 13.44 ^a^	13.97 ± 0.75 ^abc^	0.30 ± 0.03 ^cd^
	Savoury (1.0%)	71.75 ± 19.34 ^a^	13.60 ± 0.30 ^ab^	0.27 ± 0.01 ^ab^
	Fennel (1.0%)	61.71 ± 6.98 ^a^	14.23 ± 0.55 ^bc^	0.26 ± 0.01 ^ab^
	Basil (1.0%)	70.63 ±11.43 ^a^	15.00 ± 0.35 ^c^	0.37 ± 0.02 ^e^
	Oregano (1.0%)	65.70 ± 19.54 ^a^	15.10 ± 0.36 ^c^	0.28 ± 0.00 ^bc^

* Values are expressed as the mean of 3 replicates with 4 fruits ± standard deviation (SD).

## Data Availability

Not applicable.

## Supplementary Materials

The following supporting information can be downloaded at: https://www.mdpi.com/article/10.3390/ijms24032900/s1.

## References

[1] Rossi R., European Parliamentary Research Service (2019). The EU Fruit and Vegetable Sector: Main Features, Challenges and Prospects. https://www.europarl.europa.eu/thinktank/mt/document/EPRS_BRI(2019)635563.

[2] CBI Ministry of Foreign Affairs (2021). The European Market Potential for Apples. https://www.cbi.eu/market-information/fresh-fruit-vegetables/apples/market-potential.

[3] Food and Agriculture Organization of the United Nations (2020). FAOSTAT Statistical Database. https://www.fao.org/faostat/en/.

[4] Luciano-Rosario D., Keller N.P., Jurick W.M. (2020). *Penicillium expansum*: Biology, Omics, and Management Tools for a Global Postharvest Pathogen Causing Blue Mould of Pome Fruit. Mol. Plant Pathol..

[5] Quaglia M., Ederli L., Pasqualini S., Zazzerini A. (2011). Biological Control Agents and Chemical Inducers of Resistance for Postharvest Control of *Penicillium expansum* Link. on Apple Fruit. Postharvest Biol. Technol..

[6] Garello M., Piombo E., Prencipe S., Schiavon G., Berra L., Wisniewski M., Droby S., Spadaro D. (2023). Fruit microbiome: A powerful tool to study the epidemiology of dry lenticel rot and white haze—Emerging postharvest diseases of apple. Postharvest Biol. Technol..

[7] Franco Ortega S., Prencipe S., Gullino M.L., Spadaro D. (2020). New molecular tool for a quick and easy detection of apple scab in the field. Agronomy.

[8] Prencipe S., Valente S., Nari L., Spadaro D. (2022). A quantitative real-time PCR assay for early detection and quantification of *Ramularia mali*, an emerging pathogen of apple causing dry lenticel rot. Plant Dis..

[9] Spadaro D., Torres R., Errampalli D., Everett K., Ramos L., Mari M., Palou L., Smilanick J.L. (2019). Postharvest Diseases of Pome Fruit. Postharvest Pathology of Fresh Horticultural Produce.

[10] Yaseen T., Ricelli A., Turan B., Albanese P., D’Onghia A.M. (2015). Ozone for Post-Harvest Treatment of Apple Fruits. Phytopathol. Mediterr..

[11] Morales H., Marín S., Ramos A.J., Sanchis V. (2010). Influence of Post-Harvest Technologies Applied during Cold Storage of Apples in *Penicillium Expansum* Growth and Patulin Accumulation: A Review. Food Control.

[12] Reddy K.R.N., Spadaro D., Lore A., Gullino M.L., Garibaldi A. (2010). Potential of Patulin Production by *Penicillium Expansum* Strains on Various Fruits. Mycotoxin Res..

[13] Tannous J., Keller N.P., Atoui A., el Khoury A., Lteif R., Oswald I.P., Puel O. (2018). Secondary Metabolism in *Penicillium Expansum*: Emphasis on Recent Advances in Patulin Research. Crit. Rev. Food Sci. Nutr..

[14] Touhami N., Soukup S.T., Schmidt-Heydt M., Kulling S.E., Geisen R. (2018). Citrinin as an Accessory Establishment Factor of *P. Expansum* for the Colonization of Apples. Int. J. Food Microbiol..

[15] Spadaro D., Ciavorella A., Frati S., Garibaldi A., Gullino M.L. (2007). Incidence and Level of Patulin Contamination in Pure and Mixed Apple Juices Marketed in Italy. Food Control.

[16] Bräse S., Encinas A., Keck J., Nising C.F. (2009). Chemistry and Biology of Mycotoxins and Related Fungal Metabolites. Chem. Rev..

[17] Reverberi M., Ricelli A., Zjalic S., Fabbri A.A., Fanelli C. (2010). Natural Functions of Mycotoxins and Control of Their Biosynthesis in Fungi. Appl. Microbiol. Biotechnol..

[18] Andersen B., Smedsgaard J., Frisvad J.C. (2004). *Penicillium expansum*: Consistent Production of Patulin, Chaetoglobosins, and Other Secondary Metabolites in Culture and Their Natural Occurrence in Fruit Products. J. Agric. Food Chem..

[19] Morales H., Marín S., Centelles X., Ramos A.J., Sanchis V. (2007). Cold and Ambient Deck Storage Prior to Processing as a Critical Control Point for Patulin Accumulation. Int. J. Food Microbiol..

[20] Jackson L.S., Beacham-Bowden T., Keller S.E., Adhikari C., Taylor K.T., Chirtel S.J., Merker R.I. (2003). Apple Quality, Storage, and Washing Treatments Affect Patulin Levels in Apple Cider. J. Food Prot..

[21] Pitt J.I., Hocking A.D. (1997). Fungi and Food Spoilage.

[22] Morales H., Marín S., Obea L., Patiño B., Doménech M., Ramos A.J., Sanchis V. (2008). Ecophysiological Characterization of *Penicillium Expansum* Population in Lleida (Spain). Int. J. Food Microbiol..

[23] Baraldi E., Mari M., Chierici E., Pondrelli M., Bertolini P., Pratella G.C. (2003). Studies on Thiabendazole Resistance of *Penicillium Expansum* of Pears: Pathogenic Fitness and Genetic Characterization. Plant Pathol..

[24] Cabañas R., Abarca M.L., Bragulat M.R., Cabañes F.J. (2009). Comparison of Methods to Detect Resistance of *Penicillium Expansum* to Thiabendazole. Lett. Appl. Microbiol..

[25] Eckert J.W., Ogawa J.M. (1988). The Chemical Control of Postharvest Diseases: Deciduous Fruits, Berries, Vegetables and Root/Tuber Crops. Annu. Rev. Phytopathol..

[26] Ippolito A., Nigro F. (2000). Impact of Preharvest Application of Biological Control Agents on Postharvest Diseases of Fresh Fruits and Vegetables. Crop Prot..

[27] Etebarian H.R., Sholberg P.L., Eastwell K.C., Sayler R.J. (2005). Biological Control of Apple Blue Mold with *Pseudomonas Fluorescens*. Can. J. Microbiol..

[28] Calvo J., Calvente V., de Orellano M.E., Benuzzi D., Sanz de Tosetti M.I. (2007). Biological Control of Postharvest Spoilage Caused by *Penicillium Expansum* and *Botrytis Cinerea* in Apple by Using the Bacterium *Rahnella Aquatilis*. Int. J. Food Microbiol..

[29] Moodley R.S., Govinden R., Odhav B. (2002). The Effect of Modified Atmospheres and Packaging on Patulin Production in Apples. J. Food Prot..

[30] Baert K., Devlieghere F., Bo L., Debevere J., de Meulenaer B. (2008). The Effect of Inoculum Size on the Growth of Penicillium Expansum in Apples. Food Microbiol..

[31] Chung H.-S., Moon K.-D., Chung S.-K., Choi J.-U. (2005). Control of Internal Browning and Quality Improvement of ‘Fuji’ Apples by Stepwise Increase of CO2 Level during Controlled Atmosphere Storage. J. Sci. Food Agric..

[32] Sitton J.W. (1992). Effect of High-Carbon Dioxide and Low-Oxygen Controlled Atmospheres on Postharvest Decays of Apples. Plant Dis..

[33] Lopez-Reyes J.G., Spadaro D., Gullino M.L., Garibaldi A. (2010). Efficacy of Plant Essential Oils on Postharvest Control of Rot Caused by Fungi on Four Cultivars of Apples in Vivo. Flavour Fragr. J..

[34] Santoro K., Maghenzani M., Chiabrando V., Bosio P., Gullino M.L., Spadaro D., Giacalone G. (2018). Thyme and Savory Essential Oil Vapor Treatments Control Brown Rot and Improve the Storage Quality of Peaches and Nectarines, but Could Favor Gray Mold. Foods.

[35] Schiavon G., Garello M., Prencipe S., Meloni G.R., Buonsenso F., Spadaro D. (2023). Essential oils reduce grey mould rot of apples and modify the fruit microbiome during postharvest storage. J. Fungi.

[36] Burt S. (2004). Essential Oils: Their Antibacterial Properties and Potential Applications in Foods—A Review. Int. J. Food Microbiol..

[37] Lopez-Reyes J.G., Spadaro D., Prelle A., Garibaldi A., Gullino M.L. (2013). Efficacy of Plant Essential Oils on Postharvest Control of Rots Caused by Fungi on Different Stone Fruits In Vivo. J. Food Prot..

[38] Mari M., Bautista-Baños S., Sivakumar D. (2016). Decay Control in the Postharvest System: Role of Microbial and Plant Volatile Organic Compounds. Postharvest Biol. Technol..

[39] Bhavaniramya S., Vishnupriya S., Al-Aboody M.S., Vijayakumar R., Baskaran D. (2019). Role of essential oils in food safety: Antimicrobial and antioxidant applications. Grain Oil Sci. Technol..

[40] Barrera-Necha L.L., Bautista-Banos S., Flores-Moc H.E., Estudillo A.R. (2008). Efficacy of Essential Oils on the Conidial Germination, Growth of *Colletotrichum Gloeosporioides* (Penz.) Penz. and Sacc and Control of Postharvest Diseases in Papaya (*Carica Papaya* L.). Plant Pathol. J..

[41] Daferera D.J., Ziogas B.N., Polissiou M.G. (2000). GC-MS Analysis of Essential Oils from Some Greek Aromatic Plants and Their Fungitoxicity on Penicillium Digitatum. J. Agric. Food Chem..

[42] Elshafie H.S., Camele I. (2016). Investigating the Effects of Plant Essential Oils on Post-Harvest Fruit Decay. Fungal Pathogenicity.

[43] Marandi R.J., Hassani A., Ghosta Y., Abdollahi A., Pirzad A., Sefidkon F. (2011). Control of *Penicillium expansum* and *Botrytis cinerea* on pear with *Thymus kotschyanus*, *Ocimum basilicum* and *Rosmarinus officinalis* essential oils. J. Med. Plant Res..

[44] Lazar-Baker E.E., Hetherington S.D., Ku V.V., Newman S.M. (2011). Evaluation of Commercial Essential Oil Samples on the Growth of Postharvest Pathogen *Monilinia Fructicola* (*G. Winter*) Honey. Lett. Appl. Microbiol..

[45] Šegvić Klarić M., Kosalec I., Mastelić J., Piecková E., Pepeljnak S. (2007). Antifungal Activity of Thyme (*Thymus Vulgaris* L.) Essential Oil and Thymol against Moulds from Damp Dwellings. Lett. Appl. Microbiol..

[46] Sarkhosh A., Vargas A.I., Schaffer B., Palmateer A.J., Lopez P., Soleymani A., Farzaneh M. (2017). Postharvest Management of Anthracnose in Avocado (*Persea Americana* Mill.) Fruit with Plant-Extracted Oils. Food Packag. Shelf Life.

[47] Zhou T., Wang X., Ye B., Shi L., Bai X., Lai T. (2018). Effects of essential oil decanal on growth and transcriptome of the postharvest fungal pathogen Penicillium expansum. Postharvest Biol. Technol..

[48] Droby S., Eick A., Macarisin D., Cohen L., Rafael G., Stange R., McColum G., Dudai N., Nasser A., Wisniewski M. (2008). Role of citrus volatiles in host recognition, germination and growth of Penicillium digitatum and Penicillium italicum. Postharvest Biol. Technol..

[49] Perdones A., Sánchez-González L., Chiralt A., Vargas M. (2012). Effect of chitosan-lemon essential oil coatings on storage-keeping quality of strawberry. Postharvest Biol. Technol..

[50] Montero-Prado P., Rodriguez-Lafuente A., Nerin C. (2011). Active label-based packaging to extend the shelf-life of “Calanda” peach fruit: Changes in fruit quality and enzymatic activity. Postharvest Biol. Technol..

[51] Martínez K., Ortiz M., Albis A., Gilma Gutiérrez Castañeda C., Valencia M.E., Grande Tovar C.D. (2018). The Effect of Edible Chitosan Coatings Incorporated with Thymus capitatus Essential Oil on the Shelf-Life of Strawberry (*Fragaria x ananassa*) during Cold Storage. Biomolecules.

[52] Žebeljana A., Vicoa I., Duduka N., Žibernab B., Urbanek Krajnc A. (2019). Dynamic changes in common metabolites and antioxidants during *Penicillium expansum*-apple fruit interactions. Physiol. Mol. Plant Pathol..

[53] Beauvoit B., Belouah I., Bertin N., Cakpo C.B., Colombié S., Dai Z., Gautier H., Génard M., Moing A., Roch L. (2018). Putting primary metabolism into perspective to obtain better fruits. Ann. Bot..

[54] Neri F., Mari M., Brigati S. (2006). Control of *Penicillium expansum* by plant volatile compounds. Plant Pathol..

[55] Venturini M.E., Blanco D., Oria R. (2002). In vitro antifungal activity of several antimicrobial compounds against *Penicillium expansum*. J. Food Prot..

[56] Amorati R., Foti M.C., Valgimigli L. (2013). Antioxidant Activity of Essential Oils. J. Agric. Food Chem..

[57] Foti M.C. (2007). Antioxidant properties of phenols. J. Pharm. Pharmacol..

[58] Sharopov F.S., Wink M., Setzer W.N. (2015). Radical scavenging and antioxidant activities of essential oil components—An experimental and computational investigation. Nat. Prod. Commun..

[59] Shahat A.A., Ibrahim A.Y., Hendawy S.F., Omer E.A., Hammouda F.M., Abdel-Rahman F.H., Saleh M.A. (2011). Chemical composition, antimicrobial and antioxidant activities of essential oils from organically cultivated fennel cultivars. Molecules.

[60] da Silva B.D., Campos Bernardes P., Fontes Pinheiro P., Fantuzzi E., Roberto C.D. (2021). Chemical composition, extraction sources and action mechanisms of essential oils: Natural preservative and limitations of use in meat products. Meat Sci..

[61] Maurya A., Prasad J., Das S., Dwivedy A.K. (2021). Essential Oils and Their Application in Food Safety. Front. Sustain. Food Syst..

[62] Chen P.M., Varga D.M., Mielke E.A., Facteau T.J., Drake S.R. (1990). Control of superficial scald on “*Anjou*” pears by ethoxyquin: Oxidation of a-Farnesene and its inhibition. J. Food Sci..

[63] Lurie S., Watkins C.B. (2012). Superficial scald, its etiology and control. Postharvest Biol. Technol..

[64] Whitaker B.D. (2007). Oxidation Products of α-Farnesene Associated with Superficial Scald Development in d’Anjou Pear Fruits Are Conjugated Trienols. Agric. Food Chem..

[65] Dias C., Amaro A.L., Salvador Â.C., Silvestre A.J.D., Rocha S.M., Isidoro N., Pintado M. (2020). Strategies to Preserve Postharvest Quality of Horticultural Crops and Superficial Scald Control: From Diphenylamine Antioxidant Usage to More Recent Approaches. Antioxidants.

[66] Rowan D.D., Hunt M.B., Fielder S., Norris J., Sherburn M.S. (2001). Conjugated Triene Oxidation Products of α-Farnesene Induce Symptoms of Superficial Scald on Stored Apples. J. Agric. Food Chem..

[67] Souleyre E.J.F., Bowen J.K., Matich A.J., Tomes S., Chen X., Hunt M.B., Wang M.Y., Ileperuma N.R., Richards K., Rowan D.D. (2019). Genetic control of α-farnesene production in apple fruit and its role in fungal pathogenesis. Plant J..

[68] Gong D., Bi Y., Zong Y., Li Y., Sionov E., Prusky D. (2022). Characterization and sources of volatile organic compounds produced by postharvest pathogenic fungi colonized fruit. Postharvest Biol. Technol..

[69] Marchese A., Arciola C.R., Coppo E., Barbieri R., Barreca D., Chebaibi S., Sobarzo-Sánchez E., Fazel Nabavi S., Mohammad Nabavi S., Daglia M. (2018). The natural plant compound carvacrol as an antimicrobial and anti-biofilm agent: Mechanisms, synergies and bio-inspired anti-infective materials. Biofouling.

