# Supplementary material for: Efficacy and Mechanisms of Action of Essential Oils’ Vapours against Blue Mould on Apples Caused by Penicillium expansum
Source: Int J Mol Sci. 2023 Feb 2;24(3):2900. doi: 10.3390/ijms24032900 (PMC9917833; doi:10.3390/ijms24032900)
Supplement: Supplementary file 1 [file ijms-24-02900-s001.zip › ijms-2176049-supplementary.pdf]

Supplementary Materials

Efficacy and mechanisms of action of essential oils' vapours against blue mould on apples caused by *Penicillium expansum*

Fabio Buonsenso [a,b], Giada Schiavon [a,b] and Davide Spadaro [a,b,*]

[a] Department of Agricultural, Forestry and Food Sciences (DISAFA), University of Turin, Largo Paolo Braccini 2, 10095 Grugliasco (Italy)

[b] Centre of Competence for the Innovation in the Agro-environmental Sector - AGROINNOVA, University of Turin, Largo Paolo Braccini 2, 10095 Grugliasco (Italy)

*Corresponding author; email address: davide.spadaro@unito.it

Table S1. Percentage composition of EOs and retention times (r.t.) of the single molecules.

		**Basil**	**Oregano**	**Savoury**	**Thyme**	**Lemon**	**Fennel**
**Compound**	**r.t. (min)**	%	%	%	%	%	%
Unknown	5.25	-	-	-	0.08	-	-
α-Thujene	5.35	0.04	1.37	1.03	0.85	0.46	0.08
α-Pinene	5.54	0.42	1.09	1.63	1.22	2.13	14.41
Camphene	6.01	0.08	0.14	0.36	1.40	0.08	0.27
Sabinene	6.75	0.47	-	-	-	2.15	0.27
β-Pinene	6.89	0.88	0.62	1.12	0.61	13.09	1.17
3-Octanone	7.15	-	0.24	0.17	0.14	-	-
β-Mircene	7.31	0.81	2.07	1.66	1.70	1.61	1.21
Unknown	7.47	-	-	0.07	0.07	-	-
α-Phellandrene	7.87	-	0.28	0.25	0.24	-	6.28
4-Carene	8.11	-	0.08	0.12	0.11	-	-
Terpinolene	8.36	0.10	1.57	1.36	1.48	0.09	-
p-Cymene	8.65	0.23	8.04	18.17	18.95	1.44	1.53
Limonene	8.83	0.63	0.63	0.99	0.88	66.92	2.72
Eucalyptol	8.94	9.67	0.14	0.16	0.11	0.13	0.11
Unknown	9.20	-	-	0.16	-	-	0.22
α-Fenchene	9.66	0.31	0.11	0.12	-	0.12	-
γ-Terpinene	10.20	0.06	7.37	14.61	9.91	8.84	0.69
3-Carene	10.57	0.16	0.39	0.32	0.31	-	0.07
Fenchone	11.53	0.13	0.14	0.18	0.33	0.30	16.96
Linalool	12.23	58.28	2.24	1.75	5.73	0.15	0.16
Unknown	12.51	-	-	-	0.23	-	-
Camphor	14.51	0.82	0.02	0.11	1.00	-	0.33
Borneol	15.79	0.43	0.14	1.55	2.37	-	-
Terpinen-4-ol	16.41	0.70	0.76	0.69	1.79	-	-

α-Terpineol	17.20	1.10	0.01	0.17	0.38	0.14	-	
p-Allylanisole (Estragole)	17.58	0.94	0.01	0.04	-	-	1.84	
Umbellulol	17.81	-	-	-	-	-	0.21	
Octyl formate	18.56	0.32	-	-	-	-	-	
Citral	20.28 + 22.13	-	-	-	-	1.57	-	
O-Methylthymol	20.45	-	-	2.95	0.30	-	-	
p-Anisaldehyde	20.93	-	-	-	-	-	0.80	
Unknown	21.20	-	-	0.10	-	-	-	
trans-Anethole	23.01	1.16	-	-	-	-	50.45	
Thymol	23.61	-	1.98	4.12	43.26	-	-	
Carvacrol	24.31	-	68.00	41.45	4.43	-	-	
Isothujol	24.86	-	-	-	-	-	0.24	
Santolina triene	26.22	0.36	-	-	-	-	-	
Limonene epoxide	26.58	0.13	-	-	-	-	-	
Germacrene A	27.01	0.10	-	-	-	-	-	
Eugenol	27.50	2.42	-	-	-	-	-	
α-Cubebene	28.64	0.19	-	-	-	0.40	-	
α-Bourbonene	29.17	0.53	-	-	-	-	-	
β-Elemene	29.68	3.28	-	-	-	-	-	
β-Caryophyllene	31.36	0.85	2.05	4.00	2.11	-	-	
trans-α-Bergamotene	32.44	5.75	-	-	-	-	-	
α-Guaiene	32.56	0.57	-	-	-	-	-	
β-Ocimene	33.40	1.16	0.27	0.07	-	-	-	
Germacrene D	35.11	1.01	-	-	-	-	-	
Aromandrene	35.41	0.46	-	-	-	-	-	
Cis-α-Bisabolene	36.10	0.77	-	-	-	-	-	
α-Bulsene	36.68	1.05	-	-	-	-	-	
Unknown	37.01	-	0.22	0.43	-	-	-	
α-Farnesene	37.15	2.15	-	-	-	0.37	-	
Unknown	40.26	0.27	-	-	-	-	-	
Unknown	41.24	-	0.04	0.08	-	-	-	
Cedrene	43.11	0.30	-	-	-	-	-	
Sativene	44.71	0.94	-	-	-	-	-	

Table S2: Volatile organic compounds in the control cabinets at 1, 10, 28, 43 and 50 days of storage.

Control		Temperature: 1±1 °C								15±1 °C	
		t1		t10		t28		t43		t50	
**Compound**	**Retention time (min)**	%	ppm	%	ppm	%	ppm	%	ppm	%	ppm
Hexyl acetate	8.10	23.40	6.85	46.03	23.24	46.33	23.69	49.17	34.77	17.41	27.35
Hexyl butyrate	17.30	13.00	3.81	13.71	6.92	15.69	8.02	17.01	12.03	16.32	25.64
Hexyl 2-methylbutanoate	20.20	13.30	3.90	9.74	4.92	10.17	5.20	10.38	7.34	12.65	19.88
Hexyl hexanoate	29.50	9.77	2.86	8.56	4.32	6.60	3.38	6.50	4.60	12.06	18.94
α-Farnesene	37.15	39.44	11.55	20.66	10.43	17.04	8.71	16.05	11.35	37.25	58.52
2,2,4-Trimethyl-3-(carboxyisopropyl)pentanoic acid isobutyl ester	42.64	0.00	0.00	0.00	0.00	0.00	0.00	0.00	0.00	4.10	6.44
2,6,10,14-tetramethyl pentadecane	44.50	1.09	0.32	1.30	0.66	4.18	2.14	0.89	0.63	0.22	0.34
4-sec-Butyl-2,6-di-tert-butylphenol	44.78	0.00	0.00	0.00	0.00	0.00	0.00	0.00	0.00	0.00	0.00

Table S3: Volatile organic compounds in the cabinets of inoculated control fruit at 1, 10, 28, 43 and 50 days of storage.

Inoculated control		Temperature: 1±1 °C								15±1 °C	
		t1		t10		t28		t43		t50	
**Compound**	**Retention time (min)**	%	ppm	%	ppm	%	ppm	%	ppm	%	ppm
Hexyl acetate	8.10	22.81	7.23	34.41	27.71	40.29	34.29	56.13	46.65	23.71	29.81
Hexyl butyrate	17.30	12.21	3.87	9.97	8.03	12.55	10.68	9.99	8.31	11.89	14.95
Hexyl 2-methylbutanoate	20.20	16.48	5.22	11.06	8.90	12.16	10.35	8.95	7.44	17.63	22.17
Hexyl hexanoate	29.50	8.59	2.72	6.59	5.31	7.52	6.40	5.53	4.60	10.27	12.91
α-Farnesene	37.15	18.69	5.92	13.63	10.98	15.48	13.17	10.03	8.33	31.01	38.99

2,2,4-Trimethyl-3-(carboxyisopropyl)pentanoic acid isobutyl ester	42.64	18.29	5.79	21.52	17.33	9.52	8.10	2.66	2.21	5.04	6.33
2,6,10,14-tetramethyl pentadecane	44.50	0.75	0.24	1.03	0.83	0.77	0.66	6.69	5.56	0.45	0.57
4-sec-Butyl-2,6-di-tert-butylphenol	44.78	2.17	0.69	1.79	1.44	1.72	1.46	0.02	0.02	0.00	0.00

Table S4: Basil essential oil composition and **v**olatile organic compounds in the cabinets of fruit treated with basil essential oil at 1.0 % at 1, 10, 28, 43 and 50 days of storage.

**Basil EO 1.0 %**			**Temperature: 1±1 °C**								**15±1 °C**	
**Compound**	**%**	**Retention time (min)**	**t1**		**t10**		**t28**		**t43**		**t50**	
			%	ppm	%	ppm	%	ppm	%	ppm	%	ppm
α-Thujene	0.04	5.35	0.00	0.00	0.05	0.03	0.00	0.00	0.00	0.00	0.00	0.00
α-Pinene	0.42	5.54	0.00	0.00	0.00	0.00	0.00	0.00	0.00	0.00	0.00	0.00
Camphene	0.08	6.01	0.00	0.00	0.00	0.00	0.00	0.00	0.00	0.00	0.00	0.00
Sabinene	0.47	6.75	0.00	0.00	0.00	0.00	0.00	0.00	0.00	0.00	0.00	0.00
β-Pinene	0.88	6.89	0.00	0.00	0.00	0.00	0.00	0.00	0.00	0.00	0.00	0.00
β-Mircene	0.81	7.31	0.00	0.00	1.21	0.81	0.88	0.43	1.40	0.61	0.75	0.45
Hexyl acetate	-	8.10	3.45	2.58	16.58	11.11	13.72	6.76	22.52	9.87	4.09	2.46
Terpinolene	0.10	8.36	0.00	0.00	0.00	0.00	0.00	0.00	0.00	0.00	0.00	0.00
p-Cymene	0.23	8.65	1.13	1.97	2.84	4.44	0.86	0.99	1.69	1.72	0.67	0.95
Limonene	0.63	8.83	0.00	0.00	0.00	0.00	0.00	0.00	0.00	0.00	0.00	0.00
Eucalyptol	9.67	8.94	0.96	0.72	1.40	0.94	0.86	0.42	1.68	0.74	0.67	0.40
α-Fenchene	0.31	9.66	0.00	0.00	0.00	0.00	0.00	0.00	0.00	0.00	0.00	0.00
γ-Terpinene	0.06	10.20	0.10	0.08	0.18	0.12	0.00	0.00	0.00	0.00	0.13	0.08
3-Carene	0.16	10.57	0.23	0.17	0.30	0.20	0.47	0.23	0.18	0.08	0.72	0.43
Fenchone	0.13	11.53	0.30	0.22	0.15	0.10	0.27	0.13	0.13	0.06	0.56	0.34
Linalool	58.28	12.23	57.52	42.97	32.01	21.45	19.72	9.71	24.98	10.95	18.74	11.30

Camphor	0.82	14.51	0.51	0.38	0.56	0.38	0.05	0.03	0.00	0.00	0.34	0.20
Borneol	0.43	15.79	0.51	0.38	0.62	0.41	0.12	0.06	0.00	0.00	0.47	0.28
Terpinen-4-ol	0.70	16.41	1.25	0.94	0.76	0.51	0.33	0.16	0.01	0.01	0.62	0.37
α-Terpineol	1.10	17.20	4.50	3.36	4.72	3.16	4.45	2.19	6.01	2.63	0.28	0.17
Hexyl butyrate	-	17.30	4.48	3.35	4.72	3.16	4.45	2.19	6.01	2.63	6.31	3.80
p-Allylanisole (Estragole)	0.94	17.58	1.65	1.24	0.98	0.66	0.54	0.27	1.29	0.56	1.55	0.93
Octyl formate	0.32	18.56	0.45	0.34	0.02	0.02	0.25	0.12	0.10	0.04	0.12	0.07
Hexyl 2-methylbutanoate	-	20.20	2.56	1.92	4.56	3.06	4.66	2.29	6.41	2.81	12.17	7.34
t-Anethole	1.16	23.01	0.50	0.99	0.44	0.78	0.48	0.64	0.70	0.82	0.84	1.36
Santolina triene	0.36	26.22	0.17	0.13	0.02	0.01	0.08	0.04	0.33	0.15	0.25	0.15
Limonene epoxide	0.13	26.58	0.30	0.22	0.37	0.25	0.00	0.00	0.11	0.05	0.00	0.00
Germacrene A	0.10	27.01	0.15	0.11	0.03	0.02	0.00	0.00	0.13	0.06	0.00	0.00
Eugenol	2.42	27.50	1.69	1.26	3.37	2.26	4.24	2.09	0.78	0.34	1.12	0.68
α-Cubebene	0.19	28.64	0.13	0.10	4.72	3.16	3.23	1.59	0.43	0.19	0.00	0.00
α-Bourbonene	0.53	29.17	0.29	0.22	0.82	0.55	0.76	0.37	0.10	0.04	0.00	0.00
Hexyl hexanoate	-	29.50	1.45	1.09	2.69	1.80	2.84	1.40	2.89	1.26	5.62	3.39
β-Elemene	3.28	29.68	1.05	0.79	0.73	0.49	0.84	0.41	0.69	0.30	1.60	0.97
β-Caryophyllene	0.85	31.36	0.34	0.25	0.32	0.22	0.34	0.17	0.39	0.17	0.86	0.52
trans-α-Bergamotene	5.75	32.44	1.60	1.19	0.94	0.63	2.01	0.99	1.00	0.44	2.24	1.35
α-Guaiene	0.57	32.56	0.00	0.00	0.07	0.05	2.54	1.25	0.00	0.00	0.00	0.00
β-Ocimene	1.16	33.40	0.44	0.33	0.15	0.10	0.22	0.11	0.24	0.11	0.43	0.26
Germacrene D	1.01	35.11	0.17	0.13	0.14	0.09	2.72	1.34	0.24	0.10	0.11	0.07
Aromandrene	0.46	35.41	0.34	0.26	0.23	0.16	0.12	0.06	0.17	0.07	0.15	0.09
Cis-α-Bisabolene	0.77	36.10	0.39	0.29	0.57	0.38	0.56	0.28	0.10	0.04	0.21	0.13
α-Bulsene	1.05	36.68	0.35	0.26	0.35	0.23	0.29	0.14	0.09	0.04	0.12	0.07
α-Farnesene	2.15	37.15	6.37	4.76	10.43	6.99	11.70	5.76	12.09	5.30	37.06	22.35
Unknown	0.27	40.26	0.32	0.24	0.12	0.08	0.00	0.00	0.31	0.14	0.00	0.00
Cedrene	0.30	43.11	3.78	2.83	0.05	0.03	3.77	1.86	0.17	0.07	0.00	0.00
2,2,4-Trimethyl-3-(carboxyisopropyl)pentanoic acid isobutyl ester	-	42.64	0.20	0.15	0.93	0.62	9.37	4.61	5.70	2.50	0.95	0.57
2,6,10,14-tetramethyl pentadecane	-	44.50	0.00	0.00	0.00	0.00	0.00	0.00	0.00	0.00	0.00	0.00

Sativene	0.94	44.71	0.25	0.19	0.48	0.32	1.34	0.66	0.47	0.21	0.11	0.07
4-sec-Butyl-2,6-di-tert-butylphenol	-	44.78	0.11	0.08	0.39	0.26	0.92	0.45	0.45	0.20	0.10	0.06

Table S5: Oregano essential oil composition and volatile organic compounds in the cabinets of fruit treated with oregano essential oil at 1.0 % at 1, 10, 28, 43 and 50 days of storage.

Oregano EO 1.0 %			Temperature: 1±1 °C								15±1 °C	
Compound	%	Retention time (min)	t1		t10		t28		t43		t50	
			%	ppm	%	ppm	%	ppm	%	ppm	%	ppm
α-Thujene	1.37	5.35	0.00	0.00	0.00	0.00	0.00	0.00	0.00	0.00	0.00	0.00
α-Pinene	1.09	5.54	0.00	0.00	0.00	0.00	0.00	0.00	0.00	0.00	0.00	0.00
Camphene	0.14	6.01	0.00	0.00	0.00	0.00	0.00	0.00	0.00	0.00	0.00	0.00
β-Pinene	0.62	6.89	0.36	0.76	0.00	0.00	0.33	0.63	0.39	0.62	0.20	0.52
3-Octanone	0.24	7.15	0.00	0.00	0.00	0.00	0.00	0.00	0.00	0.00	0.19	0.50
β-Mircene	2.07	7.31	0.84	1.78	1.66	3.14	1.61	3.06	2.51	3.95	1.57	4.09
α-Phellandrene	0.28	7.87	0.00	0.00	0.00	0.00	0.00	0.00	0.00	0.00	0.00	0.00
Hexyl acetate	-	8.10	6.68	14.11	19.06	36.12	18.78	35.67	29.69	46.67	11.01	28.74
4-Carene	0.08	8.11	0.00	0.00	0.00	0.00	0.00	0.00	0.00	0.00	0.00	0.00
Terpinolene	1.57	8.36	1.30	2.75	2.47	4.69	2.17	4.12	4.14	6.50	0.94	2.46
p-Cymene	8.04	8.65	1.25	1.84	1.46	1.94	1.30	1.72	2.41	2.65	0.81	1.48
Limonene	0.63	8.83	1.01	1.06	0.85	0.80	0.90	0.86	1.38	1.08	0.36	0.47
Eucalyptol	0.14	8.94	0.00	0.00	0.00	0.00	0.00	0.00	0.00	0.00	0.00	0.00
α-Fenchene	0.11	9.66	0.00	0.00	0.00	0.00	0.00	0.00	0.00	0.00	0.00	0.00
γ-Terpinene	7.37	10.20	0.00	0.00	0.00	0.00	0.00	0.00	0.00	0.00	0.00	0.00
3-Carene	0.39	10.57	0.00	0.00	0.00	0.00	0.00	0.00	0.00	0.00	0.00	0.00
Fenchone	0.14	11.53	0.97	2.06	0.37	0.70	0.09	0.18	0.41	0.65	0.16	0.42
Linalool	2.24	12.23	7.92	5.02	4.10	2.33	2.86	1.63	2.81	1.33	1.82	1.38
Camphor	0.02	14.51	0.26	0.54	0.00	0.00	0.00	0.00	0.00	0.00	0.28	0.72

Borneol	0.14	15.79	1.05	2.22	0.38	0.70	0.69	1.31	0.32	0.51	0.33	0.87
Terpinen-4-ol	0.76	16.41	3.00	6.33	1.57	2.98	0.97	1.83	0.81	1.27	0.95	2.48
α-Terpineol	0.01	17.20	5.49	11.60	2.02	3.84	2.10	4.00	2.49	3.91	3.46	9.03
Hexyl butyrate	-	17.30	5.49	11.60	5.72	10.84	6.23	11.83	7.34	11.54	7.22	18.84
p-Allylanisole (Estragole)	0.01	17.58	1.76	3.72	1.76	3.34	2.10	4.00	2.49	3.91	3.46	9.03
Hexyl 2-methylbutanoate	-	20.20	5.30	11.19	4.61	8.73	4.73	8.98	5.68	8.93	9.01	23.51
O-Methylthymol	-	20.45	1.78	3.75	1.74	3.30	2.45	4.65	2.85	4.48	4.26	11.11
Thymol	1.98	23.61	2.08	1.76	1.56	1.19	1.21	0.92	0.58	0.37	0.91	0.95
Carvacrol	68.00	24.31	38.55	81.41	38.44	72.86	25.08	47.64	17.94	28.20	24.85	64.87
Hexyl hexanoate	-	29.50	4.06	8.58	3.80	7.21	4.24	8.06	4.06	6.38	8.33	21.74
β-Caryophyllene	2.05	31.36	1.21	2.55	0.16	0.30	1.28	2.42	0.69	1.09	1.11	2.89
β-Ocimene	0.27	33.40	0.00	0.00	0.00	0.00	0.00	0.00	0.00	0.00	0.16	0.42
Unknown	0.22	37.01	0.00	0.00	0.00	0.00	0.03	0.05	0.00	0.00	0.00	0.00
α-Farnesene	-	37.15	9.02	19.05	6.53	12.37	5.52	10.48	5.61	8.83	16.61	43.35
Unknown	0.04	41.24	0.00	0.00	0.00	0.00	0.03	0.05	0.00	0.00	0.00	0.00
2,2,4-Trimethyl-3-(carboxyisopropyl)pentanoic acid isobutyl ester	-	42.64	0.14	0.29	0.61	1.15	0.90	1.70	0.00	0.00	0.00	0.00
2,6,10,14-tetramethyl pentadecane	-	44.50	0.31	0.65	0.39	0.73	12.50	23.74	5.28	8.29	2.02	5.27
4-sec-Butyl-2,6-di-tert-butylphenol	-	44.78	0.18	0.37	0.75	1.41	1.97	3.74	0.12	0.19	0.01	0.04

Table S6: Savoury essential oil composition and volatile organic compounds in the cabinets of fruit treated with savoury essential oil at 1.0 % at 1, 10, 28, 43 and 50 days of storage.

**Savoury EO 1.0 %**			**Temperature: 1±1 °C**								**15±1 °C**	
**Compound**	**%**	**Retention time (min)**	**t1**		**t10**		**t28**		**t43**		**t50**	
			%	ppm	%	ppm	%	ppm	%	ppm	%	ppm
α-Thujene	1.03	5.35	0.00	0.00	0.00	0.00	0.00	0.00	0.00	0.00	0.00	0.00

α-Pinene	1.63	5.54	0.00	0.00	0.00	0.00	0.00	0.00	0.00	0.00	0.00	0.00
Camphene	0.36	6.01	0.00	0.00	0.00	0.00	0.00	0.00	0.00	0.00	0.00	0.00
β-Pinene	1.12	6.89	2.10	3.28	0.39	1.33	0.77	1.52	0.38	0.59	0.16	0.42
3-Octanone	0.17	7.15	0.36	0.56	0.21	0.72	0.42	0.83	0.26	0.41	0.39	1.03
β-Mircene	1.66	7.31	1.02	1.59	1.32	4.50	1.84	3.66	1.59	2.47	0.85	2.21
Unknown	0.07	7.47	0.38	0.59	0.00	0.00	0.00	0.00	0.00	0.00	0.00	0.00
α-Phellandrene	0.25	7.87	2.71	4.25	2.51	8.56	5.39	10.71	5.23	8.12	1.50	3.94
Hexyl acetate	-	8.10	2.77	4.34	2.21	7.53	3.03	6.01	6.30	9.79	3.44	9.01
4-Carene	0.12	8.11	0.00	0.00	0.00	0.00	0.00	0.00	0.00	0.00	0.00	0.00
Terpinolene	1.36	8.36	5.35	8.39	2.38	8.14	3.17	6.30	9.82	15.26	3.30	8.62
p-Cymene	18.17	8.65	17.19	18.86	10.20	24.36	18.61	25.88	18.31	19.91	8.63	15.82
Limonene	0.99	8.83	0.00	0.00	0.00	0.00	0.00	0.00	0.00	0.00	0.00	0.00
Eucalyptol	0.16	8.94	0.00	0.00	0.00	0.00	0.00	0.00	0.00	0.00	0.00	0.00
Unknown	0.16	9.20	0.00	0.00	0.00	0.00	0.00	0.00	0.00	0.00	0.00	0.00
α-Fenchene	0.12	9.66	0.00	0.00	0.00	0.00	0.00	0.00	0.00	0.00	0.00	0.00
γ-Terpinene	14.61	10.20	5.96	9.34	3.89	13.28	7.94	15.76	7.61	11.82	3.71	9.70
3-Carene	0.32	10.57	1.21	1.89	1.31	4.47	1.24	2.46	0.52	0.82	0.85	2.23
Fenchone	0.18	11.53	0.00	0.00	0.10	0.35	0.13	0.26	0.00	0.00	0.00	0.00
Linalool	1.75	12.23	9.92	4.67	3.60	3.69	4.83	2.88	3.11	1.45	3.43	2.70
Camphor	0.11	14.51	0.51	0.80	0.00	0.00	0.23	0.45	0.47	0.72	0.44	1.16
Borneol	1.55	15.79	4.23	6.63	2.18	7.43	2.87	5.70	2.87	4.45	2.82	7.38
Terpinen-4-ol	0.69	16.41	2.04	3.20	0.75	2.56	1.13	2.24	1.34	2.09	1.19	3.11
α-Terpineol	0.17	17.20	0.36	0.57	0.01	0.03	0.00	0.00	0.00	0.00	0.00	0.00
Hexyl butyrate	-	17.30	3.75	5.88	3.25	11.07	5.41	10.75	4.52	7.03	5.18	13.55
p-Allylanisole (Estragole)	0.04	17.58	0.53	0.83	0.01	0.03	0.64	1.28	0.86	1.33	1.26	3.29
Hexyl 2-methylbutanoate	-	20.20	3.60	5.63	2.09	7.13	4.06	8.06	4.21	6.54	7.51	19.67
O-Methylthymol	2.95	20.45	6.87	10.77	2.82	9.61	4.38	8.71	4.79	7.43	5.28	13.81
Unknown	0.10	21.20	0.24	0.37	0.09	0.32	0.01	0.03	0.12	0.18	0.27	0.70
Thymol	4.12	23.61	1.93	1.21	1.80	2.45	2.29	1.82	1.88	1.17	2.70	2.82
Carvacrol	41.45	24.31	12.55	19.67	12.18	41.55	14.45	28.70	14.92	23.18	20.39	53.37
Hexyl hexanoate	-	29.50	2.68	4.20	1.49	5.08	2.50	4.98	2.25	3.50	5.10	13.34
β-Caryophyllene	4.00	31.36	1.68	2.64	1.05	3.57	1.21	2.40	1.45	2.25	4.07	10.65

β-Ocimene	0.07	33.40	0.46	0.72	0.12	0.41	0.03	0.06	0.19	0.29	0.38	0.98
Unknown	0.43	37.01	0.00	0.00	0.00	0.00	0.03	0.05	0.00	0.00	0.00	0.00
α-Farnesene	-	37.15	6.97	10.92	4.79	16.36	6.21	12.33	6.24	9.69	15.82	41.40
Unknown	0.08	41.24	0.00	0.00	0.00	0.00	0.03	0.05	0.00	0.00	0.00	0.00
2,2,4-Trimethyl-3-(carboxyisopropyl)pentanoic acid isobutyl ester	-	42.64	1.89	2.97	36.73	125.30	3.52	6.99	0.78	1.21	1.36	3.55
2,6,10,14-tetramethyl pentadecane	-	44.50	0.44	0.68	0.00	0.00	3.64	7.24	0.00	0.00	0.00	0.00
4-sec-Butyl-2,6-di-tert-butylphenol	-	44.78	0.31	0.48	2.51	8.58	0.03	0.05	0.00	0.00	0.00	0.00

Table S7: Thyme essential oil composition and volatile organic compounds in the cabinets of fruit treated with thyme essential oil at 1.0 % at 1, 10, 28, 43 and 50 days of storage.

**Thyme EO 1.0 %**			**Temperature: 1±1 °C**								**15±1 °C**	
**Compound**	**%**	**Retention time (min)**	**t1**		**t10**		**t28**		**t43**		**t50**	
			%	ppm	%	ppm	%	ppm	%	ppm	%	ppm
Unknown	0.08	5.25	0.00	0.00	0.00	0.00	0.00	0.00	0.00	0.00	0.00	0.00
α-Thujene	0.85	5.35	0.00	0.00	0.00	0.00	0.00	0.00	0.00	0.00	0.00	0.00
α-Pinene	1.22	5.54	0.00	0.00	0.00	0.00	0.00	0.00	0.00	0.00	0.00	0.00
Camphene	1.40	6.01	0.00	0.00	0.00	0.00	0.00	0.00	0.00	0.00	0.00	0.00
β-Pinene	0.61	6.89	0.00	0.00	0.00	0.00	0.00	0.00	0.00	0.00	0.00	0.00
3-Octanone	0.14	7.15	0.11	0.08	0.20	0.13	0.09	0.09	0.48	0.22	0.09	0.07
β-Mircene	1.70	7.31	0.18	0.12	0.29	0.19	0.24	0.26	0.81	0.37	0.74	0.53
Unknown	0.07	7.47	0.24	0.16	0.16	0.10	0.17	0.18	0.40	0.18	0.48	0.35
α-Phellandrene	0.24	7.87	0.21	0.14	0.23	0.14	0.42	0.46	0.52	0.24	0.28	0.20
Hexyl acetate	-	8.10	3.82	2.58	4.26	2.73	7.23	7.79	19.15	8.81	4.88	3.50
4-Carene	0.11	8.11	0.00	0.00	0.00	0.00	0.00	0.00	0.00	0.00	0.95	0.68

Terpinolene	1.48	8.36	2.10	1.42	8.10	5.20	1.22	1.31	3.30	1.52	0.78	0.56
p-Cymene	18.95	8.65	2.06	2.44	3.89	4.36	3.03	5.71	8.47	6.82	2.58	3.23
Limonene	0.88	8.83	1.19	1.01	0.59	0.47	0.30	0.40	1.70	0.98	0.58	0.52
Eucalyptol	0.11	8.94	0.00	0.00	0.00	0.00	0.00	0.00	0.00	0.00	0.00	0.00
γ-Terpinene	9.91	10.20	0.00	0.00	0.00	0.00	0.00	0.00	0.20	0.09	0.09	0.07
3-Carene	0.31	10.57	0.00	0.00	0.00	0.00	0.03	0.04	0.27	0.12	0.35	0.25
Fenchone	0.33	11.53	0.69	0.47	0.27	0.18	0.08	0.08	0.47	0.22	0.27	0.20
Linalool	5.73	12.23	16.31	8.27	8.42	4.05	2.98	2.41	2.82	0.97	2.52	1.35
Unknown	0.23	12.51	0.00	0.00	0.00	0.00	0.00	0.00	1.37	0.63	0.73	0.52
Camphor	1.00	14.51	3.82	2.58	1.29	0.83	0.44	0.47	1.21	0.56	1.11	0.80
Borneol	2.37	15.79	6.66	4.50	4.86	3.12	1.78	1.92	2.91	1.34	3.30	2.36
Terpinen-4-ol	1.79	16.41	3.99	2.69	2.58	1.65	0.80	0.86	1.43	0.66	1.85	1.32
α-Terpineol	0.38	17.20	0.78	0.53	4.28	2.75	3.01	3.25	6.33	2.91	6.98	5.00
Hexyl butyrate	-	17.30	4.38	2.96	0.50	0.32	0.35	0.37	1.07	0.49	1.61	1.15
Hexyl 2-methylbutanoate	-	20.20	2.79	1.89	2.93	1.88	2.00	2.11	5.17	2.38	9.08	6.51
O-Methylthymol	0.30	20.45	0.36	0.24	0.28	0.18	0.25	0.27	0.93	0.43	1.54	1.10
Thymol	43.26	23.61	29.15	19.70	30.48	19.56	13.21	14.25	16.54	7.61	24.92	17.86
Carvacrol	4.43	24.31	2.94	4.96	3.30	5.30	1.53	4.12	2.19	2.51	3.31	5.93
Hexyl hexanoate	-	29.50	2.86	1.93	2.80	1.80	1.65	1.78	2.93	1.35	5.45	3.91
β-Caryophyllene	2.11	31.36	0.14	0.09	0.59	0.38	0.01	0.01	1.06	0.49	1.55	1.11
α-Farnesene	-	37.15	7.48	5.05	8.27	5.31	53.63	57.85	7.66	3.52	20.21	14.48
2,2,4-Trimethyl-3-(carboxyisopropyl)pentanoic acid isobutyl ester	-	42.64	5.72	3.87	7.40	4.75	1.96	2.12	5.15	2.37	1.40	1.01
2,6,10,14-tetramethyl pentadecane	-	44.50	1.31	0.88	3.33	2.14	3.41	3.68	5.33	2.45	2.35	1.69
4-sec-Butyl-2,6-di-tert-butylphenol	-	44.78	0.73	0.49	0.70	0.45	0.21	0.23	0.13	0.06	0.00	0.00

Table S8: Lemon essential oil composition and **v**olatile organic compounds in the cabinets of fruit treated with lemon essential oil at 1.0 % at 1, 10, 28, 43 and 50 days of storage.

**Lemon EO 1.0 %**			**Temperature: 1±1 °C**								**15±1 °C**	
**Compound**	**%**	**Retention time (min)**	**t1**		**t10**		**t28**		**t43**		**t50**	
			%	ppm	%	ppm	%	ppm	%	ppm	%	ppm
α-Thujene	0.46	5.35	0.00	0.00	0.00	0.00	0.00	0.00	0.00	0.00	0.00	0.00
α-Pinene	2.13	5.54	0.00	0.00	0.00	0.00	0.00	0.00	0.00	0.00	0.00	0.00
Camphene	0.08	6.01	0.00	0.00	0.00	0.00	0.00	0.00	0.00	0.00	0.00	0.00
Sabinene	2.15	6.75	0.00	0.00	0.00	0.00	0.00	0.00	0.24	0.04	0.42	0.16
β-Pinene	13.09	6.89	0.00	0.00	0.00	0.00	0.00	0.00	0.30	0.05	0.37	0.14
β-Mircene	1.61	7.31	0.27	0.05	0.76	0.38	2.27	0.68	1.00	0.16	1.35	0.52
Hexyl acetate	-	8.10	12.91	2.36	9.54	4.73	30.92	9.24	20.70	3.22	3.72	1.44
Terpinolene	0.09	8.36	0.00	0.00	0.00	0.00	0.00	0.00	1.90	0.30	0.95	0.37
p-Cymene	1.44	8.65	2.93	1.25	4.25	4.93	16.92	11.83	18.51	6.91	8.06	7.28
Limonene	66.92	8.83	0.00	0.00	0.00	0.00	0.00	0.00	3.18	0.85	1.64	1.06
Eucalyptol	0.13	8.94	0.00	0.00	0.00	0.00	2.13	0.64	0.00	0.00	0.14	0.05
α-Fenchene	0.12	9.66	0.00	0.00	0.00	0.00	0.40	0.12	0.00	0.00	0.22	0.09
γ-Terpinene	8.84	10.20	0.00	0.00	1.41	0.70	0.48	0.14	0.00	0.00	0.38	0.15
Fenchone	0.30	11.53	0.00	0.00	0.18	0.09	0.36	0.11	0.00	0.00	0.41	0.16
Linalool	0.15	12.23	14.52	2.66	2.84	1.41	4.05	1.21	1.21	0.19	2.19	0.85
α-Terpineol	0.14	17.20	2.35	0.43	0.25	0.12	1.10	0.33	1.65	0.26	1.61	0.62
Hexyl butyrate	-	17.30	9.88	1.81	2.27	1.13	6.41	1.91	7.35	1.14	6.77	2.63
Hexyl 2-methylbutanoate	-	20.20	15.47	2.83	3.70	1.84	6.98	2.08	9.55	1.49	15.90	6.17
Citral	1.57	20.28 + 22.13	0.25	0.04	0.08	0.04	0.10	0.03	0.11	0.02	0.06	0.02
α-Cubebene	0.40	28.64	0.00	0.00	0.76	0.37	0.47	0.14	0.00	0.00	0.00	0.00
Hexyl hexanoate	-	29.50	10.66	1.95	2.52	1.25	4.09	1.22	4.19	0.65	10.42	4.04
α-Farnesene	0.37	37.15	20.43	3.73	5.64	2.80	9.31	2.78	10.21	1.59	36.64	14.22
2,2,4-Trimethyl-3-(carboxyisopropyl)pentanoic acid isobutyl ester	-	42.64	5.02	0.92	7.41	3.68	4.64	1.39	5.08	0.79	5.50	2.14

2,6,10,14-tetramethyl pentadecane	-	44.50	4.08	0.75	53.03	26.33	9.37	2.80	14.81	2.31	3.25	1.26
4-sec-Butyl-2,6-di-tert-butylphenol	-	44.78	1.22	0.22	5.37	2.67	0.00	0.00	0.00	0.00	0.00	0.00

Table S9: Fennel essential oil composition and **v**olatile organic compounds in the cabinets of fruit treated with fennel essential oil at 1.0 % at 1, 10, 28, 43 and 50 days of storage.

**Fennel EO 1.0 %**			**Temperature: 1±1 °C**								**15±1 °C**	
**Compound**	**%**	**Retention time (min)**	**t1**		**t10**		**t28**		**t43**		**t50**	
			%	ppm	%	ppm	%	ppm	%	ppm	%	ppm
α-Thujene	0.08	5.35	0.00	0.00	0.00	0.00	0.00	0.00	0.00	0.00	0.00	0.00
α-Pinene	14.41	5.54	0.00	0.00	0.00	0.00	0.00	0.00	0.00	0.00	0.00	0.00
Camphene	0.27	6.01	0.00	0.00	0.00	0.00	0.00	0.00	0.00	0.00	0.00	0.00
Sabinene	0.27	6.75	0.00	0.00	0.00	0.00	0.00	0.00	0.00	0.00	0.00	0.00
β-Pinene	1.17	6.89	0.00	0.00	0.00	0.00	0.00	0.00	0.00	0.00	0.00	0.00
β-Mircene	1.21	7.31	1.06	0.71	1.00	1.73	0.94	0.93	0.93	0.55	1.01	0.97
α-Phellandrene	6.28	7.87	0.87	0.58	0.55	0.94	0.93	0.91	3.82	2.27	1.22	1.17
Hexyl acetate	-	8.10	11.18	7.46	8.28	14.31	16.91	16.60	14.53	8.66	2.02	1.94
p-Cymene	1.53	8.65	1.53	1.02	0.75	1.29	1.83	1.79	0.72	0.43	0.61	0.59
Limonene	2.72	8.83	0.00	0.00	0.47	0.51	0.00	0.00	0.00	0.00	0.00	0.00
Eucalyptol	0.11	8.94	0.00	0.00	0.00	0.00	0.00	0.00	0.00	0.00	0.00	0.00
Unknown	0.22	9.20	0.10	0.07	0.11	0.20	0.31	0.30	0.28	0.17	0.00	0.00
γ-Terpinene	0.69	10.20	0.00	0.00	0.00	0.00	0.00	0.00	0.00	0.00	0.00	0.00
3-Carene	0.07	10.57	0.00	0.00	0.00	0.00	0.00	0.00	0.00	0.00	0.00	0.00
Fenchone	16.96	11.53	7.20	4.80	1.68	2.90	3.43	3.37	3.81	2.27	3.30	3.17
Linalool	0.16	12.23	6.77	1.69	0.89	0.58	2.33	0.86	1.15	0.26	0.82	0.30
Camphor	0.33	14.51	0.71	0.47	0.00	0.00	0.40	0.39	0.37	0.22	0.19	0.18
Hexyl butyrate	-	17.30	7.22	4.82	4.31	7.45	8.21	8.06	7.11	4.23	4.89	4.70

p-Allylanisole (Estragole)	1.84	17.58	1.74	1.16	0.80	1.38	1.84	1.81	2.12	1.26	2.12	2.03
Umbellulol	0.21	17.81	0.91	0.61	0.34	0.59	0.65	0.64	1.01	0.60	0.61	0.59
Hexyl 2-methylbutanoate	-	20.20	8.55	5.70	3.59	6.20	9.15	8.98	9.12	5.43	12.57	12.08
p-Anisaldehyde	0.80	20.93	1.21	0.81	0.31	0.54	0.33	0.32	0.25	0.15	0.06	0.06
trans-Anethole	50.45	23.01	26.67	17.79	9.07	15.68	15.20	14.92	16.32	9.72	23.31	22.41
Isothujol	0.24	24.86	0.00	0.00	0.00	0.00	0.00	0.00	0.00	0.00	0.00	0.00
Hexyl hexanoate	-	29.50	4.63	3.09	1.76	3.04	6.59	6.46	5.12	3.05	5.45	5.24
α-Farnesene	-	37.15	13.69	9.13	6.11	10.57	18.17	17.83	17.17	10.23	36.55	35.14
2,2,4-Trimethyl-3-(carboxyisopropyl)pentanoic acid isobutyl ester	-	42.64	3.09	2.06	3.53	6.11	2.78	2.73	8.39	5.00	2.34	2.25
2,6,10,14-tetramethyl pentadecane	-	44.50	2.51	1.67	51.79	89.53	9.42	9.25	7.79	4.64	2.93	2.82
4-sec-Butyl-2,6-di-tert-butylphenol	-	44.78	0.36	0.24	4.65	8.04	0.59	0.58	0.00	0.00	0.00	0.00

Table S10: Rot incidence on 'Opal' apples treated with essential oils by biofumigation after 60 days of storage at 1±1 °C and 95% relative humidity and after 14 days of shelf life at 15±1 °C.

	**Rot incidence (%) ± SD***	
**Treatments**	**60 days of storage**	**14 days of shelf life**
Control	1 ± 0.02	9 ± 0.09
Pyrimethanil	3 ± 0.02	31 ± 0.01
Inoculated control	5 ± 0.03	41 ± 0.15
Lemon 1.0 %	3 ± 0.02	15 ± 0.11
Thyme 1.0 %	3 ± 0.01	31 ± 0.12
Savoury 1.0 %	3 ± 0.01	32 ± 0.09
Fennel 1.0 %	6 ± 0.01	43 ± 0.04
Basil 1.0 %	7 ± 0.05	29 ± 0.09
Oregano 1.0 %	1 ± 0.01	17 ± 0.05

* Values are expressed as a mean of three replicates with 4 fruits ± standard deviation (SD).
